# Structural and morphological tuning of iron oxide polymorphs by ECR plasma-assisted thermal oxidation†

**DOI:** 10.1039/d0ra05410k

**Published:** 2020-08-28

**Authors:** Supriya More, Suyog Raut, S. Premkumar, Somnath Bhopale, Sudha Bhoraskar, Mahendra More, Vikas Mathe

**Affiliations:** Department of Physics, Savitribai Phule Pune University Ganeshkhind Pune 411007 Maharashtra India vlmathe@physics.unipune.ac.in vikasmathe@gmail.com; Armament Research and Development Establishment Pune 411021 Maharashtra India

## Abstract

The work presented involves the generation of oxygen plasma species at low pressure utilizing an Electron Cyclotron Resonance (ECR) plasma reactor, and their interactions with micron- and nano-sized iron films (M-Fe and N-Fe film respectively) prepared using ethyl cellulose processed at high temperature. A specially designed radiation heater (RH) was used to raise the surface temperature of the film rapidly, exactly at the film interface, where the plasma species interact with the surface. As a result of the interaction of oxygen plasma species and temperature, iron is oxidized to different polymorphs depending on the operating pressure and hence oxygen gas flow rate. The phase, as well as the morphology of the film was controlled by monitoring the oxygen flow rate using the unique Plasma-Assisted Thermal Oxidation (PATO) process. Different polymorphs, *viz.*, Fe_3_O_4_, γ-Fe_2_O_3_, α-Fe_2_O_3_ and different morphologies, such as polygonal, compact facets, wire-like (1D) nanostructures at the surface were obtained for the films processed using PATO. The selected PATO-processed films were investigated for Field Electron Emission (FEE) properties. The 1D-grown surface of iron oxide obtained from the M-Fe film showed a turn-on field of 3 MV m^−1^ and emission current of 337 μA cm^−2^, whereas the pyramidal surface morphology obtained using N-Fe film gives a turn-on field of 3.3 MV m^−1^ with an emission current of 578 μA cm^−2^.

## Abstract 2

Schematic representation of Plasma-Assisted Thermal Oxidation (PATO)-processed M-Fe and N-Fe films with surface morphologies at optimized oxygen pressures.

## Graphical abstract

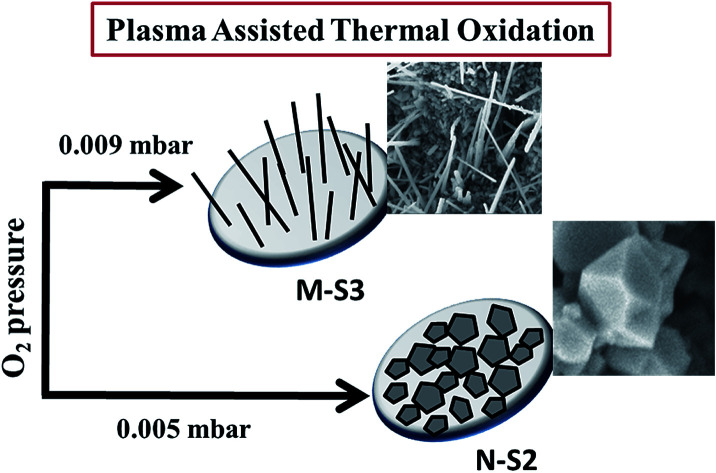

## 1. Introduction

Plasma, especially non-thermal plasma, has been used in the medical,^1^ textile^2,3^ and food processing^4–6^ industries for more than a decade for sterilization, effluent treatment, surface cleaning, *etc.* It is also used in automobile industries for surface nitridation,^7^ carbo-nitridation and carbonation of mechanical tools, also known as surface hardening. It is known that a typical type of non-thermal plasma^8^ is used, depending on the application requirement. The non-thermal plasmas, *viz.*, DC plasma,^9^ RF plasma,^10^ microwave plasma^11^ and ECR plasma, have been explored for applications like thin film deposition, surface modification, surface functionalization, surface hardening, *etc.* However, of these, the ECR plasma is relatively less explored. The ECR plasma reactor has its advantages as it operates at relatively lower pressure, higher electron density, requires no electrodes to generate plasma, and does not produce toxic gases or hazardous byproducts during the process, which make it an environmentally friendly process. The first generation of indigenously developed ECR plasma reactor by our group has been explored for applications like the surface nitridation of GaAs,^12^ En-41B steel^13^ and M2 steel^14^ using H_2_ + N_2_ (HN) plasma. The same ECR plasma reactor was used to deposit nano-crystalline diamond films.^15^ Appropriately biased hollow cathodic cylinders of Zn^16^ and Mo^17^ were introduced individually into ECR plasma to obtain oxide nanostructures and these nanostructures were further used for scanning tunneling microscopy (STM) studies. The ECR plasma source was used for the thin film deposition of Ti and Fe-doped Ti,^18^ as well as the surface modification of biocompatible polymers like poly(etherimide),^19^ suitable for tissue engineering applications.

The presently used ECR plasma reactor is a modified version of the 1^st^ generation ECR plasma reactor that was reported earlier^20^ with detailed diagnostics of the ECR plasma system for the spatial distribution of plasma properties, mainly the electron temperature (*T*_e_), plasma density (*n*_e_), *etc.*, using a Langmuir probe. Knowledge of plasma properties is quite an important factor that is used to understand the materials processing. Further, the effects of the plasma species generated using Ar, O_2_ and HN on nylon 6 have been studied.^20^ Further, the ECR plasma reactor was used to modify the surface of UHMWPE (ultra high molecular weight polyethylene) using O_2_ and HN plasma in order to study the adhesion and proliferation of bone-associated cells. The results demonstrate that the plasma treatment time was a sensitive parameter for defining the bone cell proliferation.^21^ In addition, the influence of the oxygen plasma treatment on the solar energy conversion performance of the porous ZnO-based dye-sensitized solar cells was studied.^22^ To widen the utility of the present ECR plasma reactor, the manuscript mainly focuses on the feasibility of utilizing it for materials processing, in particular, to tune the surface morphology for FEE applications. The feasibility of the earlier ECR plasma reactor was investigated using hollow cathode-biased cylinders of Mo^17^ and Ag^23^ introduced individually into the plasma reactor to obtain oxide nanostructures and further used in field emission microscopy (FEM) studies. Similarly, Kar *et al.* demonstrated the use of an ECR plasma reactor to grow carbon nanotubes on an Inconel substrate with varied process parameters to investigate the field emission behaviour.^24^

The cathode materials used in the electron guns of many devices have been a topic of interest from the viewpoint of basic understanding, as well as technological developments, for many years. To improve the performance of field emitters, nanoscale materials have been used.^10,25–31^ Nanostructured materials possess high aspect ratios, as well as high surface activity, and are, therefore, suitable for applications like field-effect electron emission, gas sensors,^32^ catalysts,^33,34^ magnetic storage devices,^35^ anode material for lithium-ion batteries,^36^ thermoelectric power generators,^37^ nuclear radiation sensors,^38^*etc.* Looking at the field emission properties of nanostructures grown on surfaces, researchers have reported that carbon nanotubes are excellent field emitters.^24,39^ However, one-dimensional (1D) materials like carbon nanotubes require prolonged treatment at high temperatures and the field emission current (performance) is degraded with time due to surface oxidation. To overcome this problem, various stable metal oxide nanomaterials like ZnO,^40^ WO_3_,^41^ CuO,^42^ and Fe_2_O_3_ ^10,25,30,43,44^ have been synthesized and used as field-effect emitters. Out of the various nano-materials, iron oxide has attracted the most attention due to its environmental friendliness, non-toxicity, excellent thermal stability, and low cost.^33,45^ Iron oxide has 3 prominent polymorphs, *viz.*, Fe_3_O_4_, γ-Fe_2_O_3_, and α-Fe_2_O_3_, of which α-Fe_2_O_3_ is the most stable under various ambient conditions, with n-type semiconductor behaviour and a band gap of 2.2 eV. These stable nanostructures have been synthesized using various methods like thermal oxidation,^30,35,46,47^ plasma oxidation,^31,48,49^ sol–gel-mediated reaction,^50^ hydrothermal reaction,^51,52^ the microwave-assisted hydrothermal method,^53–55^ template methods,^56^ chemical vapour deposition,^39^ electrochemical deposition,^28,57^ solvothermal deposition,^58,59^*etc.* It is noted from the literature that the field emission properties mainly depend on the method of synthesis used to grow the hematite phase. In some cases, post processing is required to achieve a stable hematite phase.

Table 1 summarizes the reported methods used to grow the hematite phase; more specifically, the types of methods, processing time and field emission properties. To improve the field emission performance, Junqing *et al.*^25^ reported that the sample needs additional current aging treatment, giving rise to a reduced threshold field of 6.6 MV m^−1^. Similarly, Wu *et al.*^43^ mentioned a further decrease in the threshold field to 7.2 MV m^−1^ once the samples were subjected to X-ray irradiation (a dose of 9.0 × 10^14^ phs cm^−2^). As mentioned by Liang Li,^44^ the pulse laser-deposited film needs further post-processing at 450 °C for 3 h and gives the electron emission properties as mentioned in Table 1. The synthesis process reported by Zheng *et al.*^10^ required 15 h of conventional thermal oxidation at 260 °C and further RF oxygen plasma treatment to improve the field emission properties. On the other hand, Li-Chieh *et al.*^30^ performed the conventional thermal oxidation of iron films having different thicknesses to grow the nanowires, where the substrate film thickness and growth of the nanowires per unit area defined the emission properties.

**Table 1:** Summary of the methods, processing times and field emission properties of iron oxide (α-Fe_2_O_3_) reported in the literature^a^

Sr. no.	Method	Processing time	Post process	Low turn-on field (MV m^−1^)	Threshold field (MV m^−1^)	References
1	TO	10 h	Not required	6.2 (NFs)	>11	25
2	TO	10 h	Not required	3.3 (NWs)	—	30
3	TO	10 h	Not required	5.2 (NFs)	10.1	43
4	PLD*	10 min	Required	64 (NPs)	86	44
5	TO^#^	15 h	Required	8 (NFs)	11	10

a TO: thermal oxidation, PLD: pulse laser deposition, RF: radio frequency, *: hematite column array composed of nanoparticles, #: further processing with 10 min of Ar RF plasma (NFs: nano flakes, NWs: nanowires, NP: nanoparticles).

As mentioned above, ECR plasma can produce different plasma species such as electrons, ions, atoms, molecules, *etc.*, having different energies and densities. Also, the reactivity of the ionic plasma species generated in the ECR plasma reactor is expected to be different, which is sensitive to partial pressure and temperature at the reactive sites. Therefore, in the present study, we focus on the interaction of ECR plasma species leading to the phase tuning, as well as morphology tuning, of the surface of metal films made up of micron- and nano-sized iron powders under suitable conditions. The ECR plasma being cold plasma, the interaction of plasma species with the iron surface was not seen clearly at room temperature because of the polymer coating on the precursor iron powders used during the film formation. Therefore, a radiation heater (RH) was developed to raise the surface temperature of films inside the ECR plasma reactor. The surface of the metal films was oxidized using low-pressure oxygen plasma and rapid thermal heating with the heating rate of 12 °C s^−1^. The ECR plasma produced atomic, molecular, as well as ionic oxygen species, and elevated temperature facilitated the oxidation of the iron surface. The PATO process was carried out in a closed and controlled environment. The optimized sets of films were characterized thoroughly using X-ray photoelectron spectroscopy (XPS), Raman spectroscopy, X-ray diffraction (XRD) and Field Emission-Scanning Electron Microscopy (FE-SEM). The field electron emission properties of selected films obtained by PATO were investigated.

## 2. Experimental section

### 2.1. Synthesis of iron nanoparticles using the thermal plasma route

The iron nanoparticles were synthesized using a transferred arc thermal plasma reactor. The thermal plasma reactor consists of an anode, which also acts as the sample holder for the precursor, a cathode enclosed in plasma torch, and the whole assembly was enclosed in a double-walled water-cooled plasma reactor. It was possible to vary operating pressure inside the plasma reactor using a rotary pump. The micron-sized iron particles (LOBA, ∼24 to 60 microns, purity of 99.5% electrolytic grade) in the form of pellets were kept beneath the plasma plume having a length of 5–10 cm and diameter of about 1–2 cm with high thermal flux. The operating pressure during the synthesis was maintained at 1000 torr. The iron metal species were gasified due to the sufficiently high temperature and experienced rapid thermal quenching, *i.e.*, the temperature decreased from ∼10 000 K to ∼1500 K within a 15–20 mm region from the center of the plasma plume. Due to the sharp temperature gradient, the evaporated species nucleated and grew in the plasma peripheral region. The formed nanocrystalline powder settled on the inner sides of the reactor chamber and was later scraped. The structural and morphological analysis indicated the formation of the BCC phase of iron with a maximum number of particles having a size of around 30 nm. The details of the synthesis and characterization of the iron nanoparticles were reported elsewhere.^60^

### 2.2. Preparation of M-Fe and N-Fe films

**Fig. 1:**
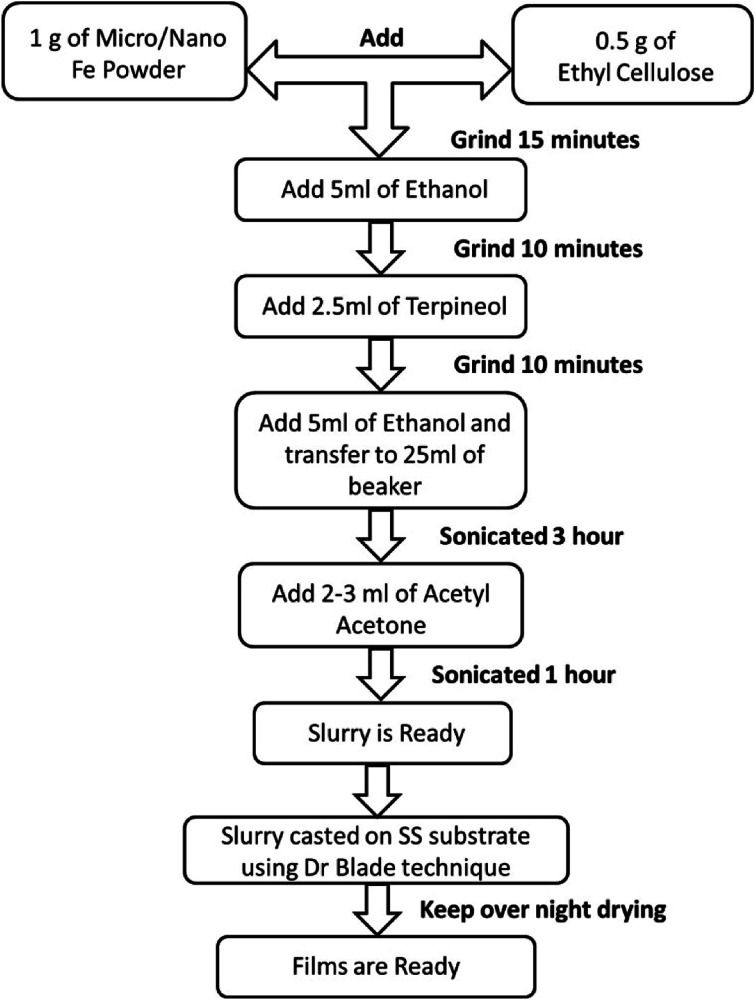
Flowchart of the process used to obtain thick films of iron in an ethylcellulose medium.

Micron-sized iron powder (M-Fe) (∼24 to 60 microns, purity of 99.5% electrolytic grade) and nano-sized iron powder synthesized using the thermal plasma process (N-Fe, average particle size ∼ 30 nm) were used as a precursor material to prepare the slurries. The process followed to prepare the slurry and thick films is shown in Fig. 1. Ethanol and acetylacetone were procured from Hayman (premium grade 100%) and Loba Chemie (99.5% pure), respectively.

The flowchart presented in Fig. 1 was followed to obtain the films. Here, ethylcellulose was used as a binder to bind the precursor particles, which facilitated the adhesion of the precursor particles to the substrate, as well as the oxidation process. The choice of ethyl cellulose as a binder is based on the reports in the literature.^61^

### 2.3. Radiation heater (RH)

**Fig. 2:**
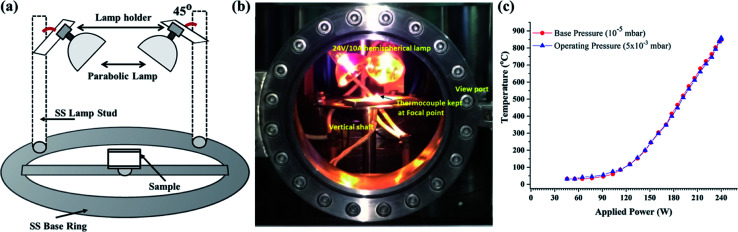
(a) Schematic representation of the radiation heater (RH) made up of a parabolic lamp arrangement mounted on an SS base inside the vacuum chamber (ECR reactor) to raise the temperature in the local region. (b) Photograph of the actual mounting of the radiation heater (RH) on a vertical shaft inside the vacuum chamber. (c) Temperature variation as a function of operating power measured at 5 × 10^−3^ mbar and 10^−5^ mbar.

The radiation heater (RH) was indigenously developed by using halogen lamps (PHILIPS 24 V/250 W, Projection lamp Type 13163). The RH consists of two lamps mounted on the circumference of the stainless steel ring of radius 10 cm. The lamps were mounted in such-a-way that the radiation was focused at the center of the ring where the film was kept. The focal point of each lamp was ∼3.5 cm away from the filament. The film was positioned (Fig. 2a) exactly at the center of the bright focal point of the lamps. The lamps were powered up using a specially designed step down transformer (24 V/10 A) connected through a dimmerstat. Fig. 2b shows the actual photograph of the RH heater assembly mounted inside the plasma reactor. The temperature profile produced by RH was recorded at the focal point as a function of the input power at the base pressure of 10^−5^ mbar and 5 × 10^−3^ mbar. Fig. 2c shows the plot of the temperature profiles obtained using RH under various operating conditions. Typically, the temperature of 850 ± 30 °C was achieved by operating both the lamps at 230 W within 1 min.

### 2.4. ECR plasma reactor

The ECR plasma reactor was used to generate the oxygen plasma species. ECR conditions were achieved by using microwave radiations of frequency 2.45 GHz and a DC magnetic field of 875 gauss. An electromagnet comprised of a pair of solenoids was used to generate the magnetic field >875 G. Before generating the plasma, the system was evacuated to a base pressure of 10^−5^ mbar using a turbo-molecular pump backed by a rotary pump and later, oxygen gas was filled in the reactor till the desired operating pressure of up to 1 to 15 × 10^−3^ mbar was reached. The resonance of the microwave field and magnetic field resulted in the formation of the glow discharge, where the ECR resonance condition was satisfied between the cyclotron motion of electrons (Lorentz force) and the input microwave frequency. The optical emission spectroscopic analysis of the oxygen ECR plasma indicated the presence of atomic oxygen species as prominent species along with other reactive species. To determine the plasma properties such as *n*_e_, *T*_e_, Debye length (*λ*_D_), *etc.*, the Langmuir probe method was used. *T*_e_ was found to vary in the range of 10–12 eV, *n*_e_ was about 10^17^ m^−3^, with *λ*_D_ of about 20–100 μm as the Langmuir Probe moved from 15 to 31 cm away from the ECR zone. Further, the Electron Energy Distribution Function (EEDF) analysis indicated that the maximum value of *T*_e_ varied in the range of 14–22 eV with an increase in the distance of the Langmuir probe from the ECR zone with a wide range of energy distribution. The detailed mapping of the ECR plasma properties was reported elsewhere.^20^ The axial mapping of the spectroscopic measurement confirmed the presence of different plasma species, *viz.*, atomic, molecular and ionic species. The M-Fe or N-Fe films were kept 23 cm away from the ECR zone, where the focal points of the RH coincide and the estimated *n*_e_ was found to be of the order of 10^17^ m^−3^ as determined using the electrostatic probe method.

### 2.5. Oxidation of iron using the plasma assisted thermal oxidation (PATO) process

Films made up of micro- and nano-sized precursor iron powders, designated respectively as M-Fe and N-Fe, were subjected to PATO. PATO was carried out in the presence of oxygen gas with different flow rates (5–200 sccm) and hence partial pressures of oxygen. The operating pressure during the PATO of M-Fe and N-Fe films was varied in the range of 1 to 15 × 10^−3^ mbar. At a given operating pressure, oxygen plasma was generated and then RH was switched ON to raise the temperature to 750 °C. The total time required to attain the desired temperature of 750 °C was about a minute with a heating rate of 12 °C s^−1^, and further oxidation was carried out for 9 min. Variation of the oxygen flow rate and hence the operating pressure at elevated temperature resulted in phase and morphological tuning. The architected iron oxide surface was used to investigate the FEE properties. The operating conditions and film designation are given in Table 2.

**Table 2:** Designation of the M-Fe and N-Fe films processed by Plasma-Assisted Thermal Oxidation (PATO) under different operating conditions^a^

Sr. no.	Material	O_2_ gas flow (sccm)	Operating pressure (mbar)	Heating time (min)	Heating rate (750 °C)	Sample name
1	M-Fe film	30	5 × 10^−3^	10	12 °C s^−1^	M-S1
2	M-Fe film	100	9 × 10^−3^	10	12 °C s^−1^	M-S2
3	M-Fe film	200	15 × 10^−3^	10	12 °C s^−1^	M-S3
4	N-Fe film	5	1 × 10^−3^	10	12 °C s^−1^	N-S0
5	N-Fe film	15	3 × 10^−3^	10	12 °C s^−1^	N-S1
6	N-Fe film	30	5 × 10^−3^	10	12 °C s^−1^	N-S2
7	N-Fe film	100	9 × 10^−3^	10	12 °C s^−1^	N-S3

a PATO: Plasma-Assisted Thermal Oxidation, sccm: standard cubic centimeter.

### 2.6. Characterization techniques

The chemical states after the PATO processing of M-Fe/N-Fe films were investigated by using the surface-sensitive X-ray Photoelectron Spectrometer (XPS, model VersaProbe III from Physical Electronics ULVAC-PH). The as-prepared M-Fe and N-Fe films were characterized for their structural properties using the X-ray diffraction (XRD) technique where a Bruker AXS D8 Advance X-ray diffractometer with Cu-Kα radiation was used to record the XRD patterns before and after the PATO process. The surface morphology of the films was investigated using Field Emission Scanning Electron Microscope (FE-SEM, Carl-Zeiss MERLIN FE-SEM). The surface-sensitive Raman spectrometer (Renishaw inVia Raman Microscope) was used to investigate the polymorphs, *viz.*, hematite, magnetite, maghemite, *etc.*, formed at the surface. A laser having a wavelength of 532 nm with a power of 0.5% was used as an excitation source and Raman spectra were recorded in the range of 100–3200 cm^−1^.

Further, the field emission properties for the morphologically tuned M-Fe and N-Fe films were investigated by using a planar diode configuration. Initially, the film of interest was fixed/stuck onto a copper rod (acts as a cathode) using carbon tape. The rod was connected to a linear motion drive, which was then used to adjust the cathode–anode separation during the field emission (FE) measurements. A typical diode configuration was used, where a semi-transparent cathodoluminescent phosphor screen (diameter ∼50 mm) was held parallel to the cathode. The FE working chamber was closed and evacuated to 1 × 10^−8^ mbar of base pressure using appropriate vacuum systems. The FE measurements were carried out at a constant cathode–anode separation of 1 mm (1000 μm), the emission current was recorded using a KEITHLEY electrometer (model 6514) by varying the applied voltage between the cathode and anode in the range of 0–40 kV (Spellman, USA), having step size of 40 V.

## 3. Results and discussion

### 3.1. Plasma-assisted thermal oxidation (PATO) process

#### 3.1.1. XPS analysis

**Fig. 3:**
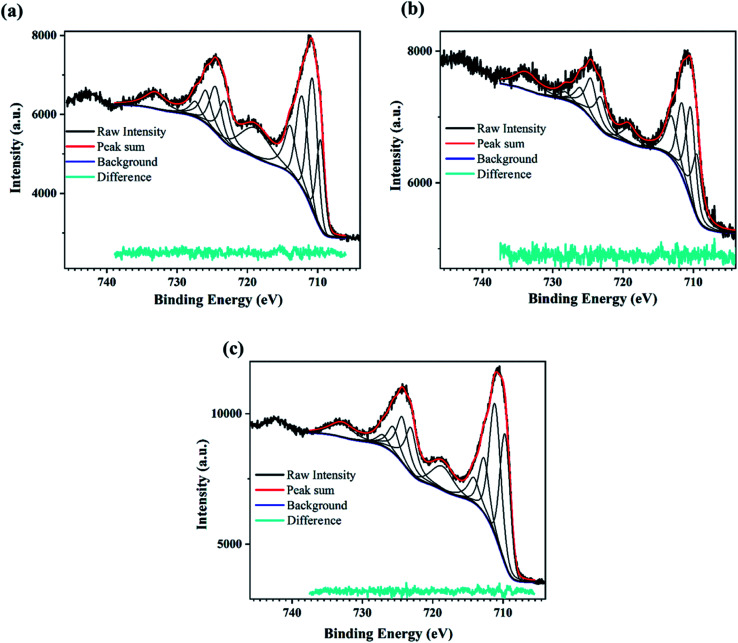
XPS scan for the PATO-processed M-Fe films showing the high-resolution peak-fitted Fe2p spectrum for the (a) M-S1, (b) M-S2 and (c) M-S3 film.

Fig. 3 shows the high-resolution XPS spectra recorded for the M-Fe oxidized films in the energy range corresponding to characteristics of Fe2p_3/2_ and Fe2p_1/2_ of iron oxide. These peaks were deconvoluted to investigate the polymorphs of iron oxide present at the surface of the M-S1 to M-S3 films. ESI Table 1† shows the summary of deconvoluted peaks corresponding to the Fe2p states and satellite peaks.

The XPS peak corresponding to Fe2p state shows two prominent peaks associated with Fe2p_3/2_ and Fe2p_1/2_. The peaks observed at around 710.7 eV and 724.5 eV correspond to Fe2p_3/2_ and Fe2p_1/2_, respectively,^62–64^ whereas broad peaks observed at 718.8 eV and 733 eV are the shake-up satellite peaks of Fe2p_3/2_ and Fe2p_1/2_, respectively.^65^ Satellites associated with the Fe2p core level spectra were used to determine the oxidation states of iron.^63^ It was mentioned by Radu *et al.*^66^ that the clearly visible satellite peak at 718 eV indicates the presence of maghemite or hematite, and the absence of the satellite peak indicates the presence of the magnetite phase.^67,68^

Fig. 3a–c shows the presence of a satellite peak (between 711 and 724 eV) at 718 eV with increasing intensity for M-S1 to M-S3 films, indicating the presence of the maghemite or hematite phase. Fujii *et al.* reported that there was hardly any difference between the XPS spectra of hematite and maghemite; however, there are certain points that can distinguish between these phases. The intensity ratio of the satellite observed at 718 eV with the main peak of Fe2p_3/2_ observed at 711 eV was less for maghemite as compared to hematite, and secondly, the peak position of 2p_3/2_ was slightly shifted towards lower binding energy in case of maghemite with respect to hematite.^67^ Therefore, to distinguish between maghemite/hematite, the XPS lines corresponding to the Fe2p spectrum needed to be carefully analyzed. From Fig. 3 and ESI Table 1,† the peak observed at 719 and 733 eV represents the signature of satellite peaks; the area under the fitted curve greatly increased from M-S1 to M-S3. The highest intensity/area of satellite peaks with the prominent Fe2p peak of the M-S3 film represents the hematite phase, confirming that the Fe^3+^ cations are octahedrally coordinated in a crystal structure.^66^ A comparison of the XPS spectra of film M-S1 and M-S3 showed that the broad peak observed at 719 eV had a very low intensity and was slightly shifted to the lower binding energy of the Fe2p_3/2_ main peak, indicating the presence of the maghemite phase according to Fujii *et al.*^67^

**Fig. 4:**
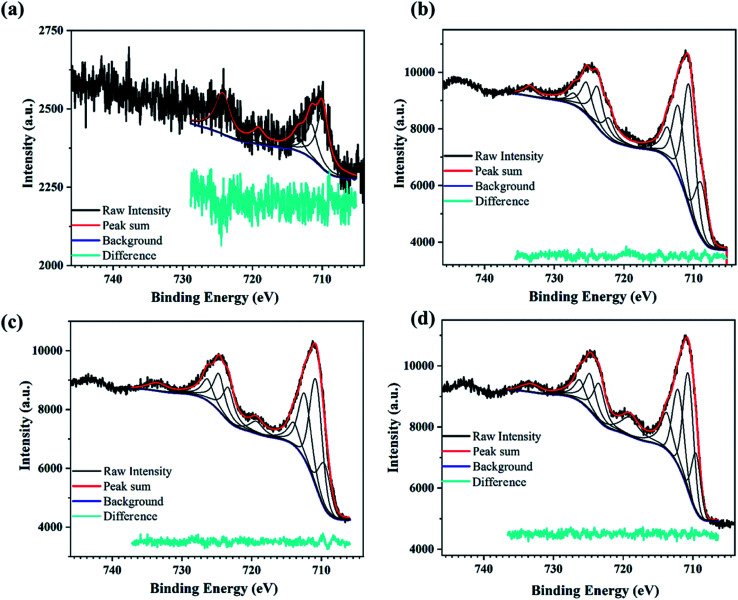
XPS spectra for PATO-processed N-Fe films showing the high-resolution peak fitted Fe2p spectrum for the (a) N-S0, (b) N-S1, (c) N-S2, and (d) N-S3 film.

The high-resolution XPS spectra recorded in the energy range corresponding to the Fe2p state for N-Fe films processed using PATO are shown in Fig. 4. The XPS fitted peak position, FWHM and the area under the curve were tabulated for the respective peaks and are shown in ESI Table 2.† The Fe2p high-resolution XPS (Fig. 4a) for the N-S0 film shows the poor signature of the 2p_3/2_ and 2p_1/2_ peak at around 710 eV and 724 eV along with the almost negligible signature of the satellite peaks, indicating the presence of the magnetite phase. Similarly, the N-S1 (Fig. 4b) film showed the prominent signature of the 2p_3/2_ and 2p_1/2_ peaks but the absence of the satellite peak at 718 eV.^57,66,67^ Further, XPS recorded for N-S2 (Fig. 4c) and N-S3 (Fig. 4d) indicated the presence of satellite peaks along with the main peaks corresponding to Fe2p_3/2_ and Fe2p_1/2_. Based on the XPS data, it was inferred that the surface polymorph of the N-S2 and N-S3 films was the hematite phase. The intensity/area under the curve of the Fe^3+^ satellite peak (719 eV) increased but that of Fe^2+^ satellite peak (733 eV) decreased in N-S3 as compared to N-S2. The N-S2 and N-S3 films have similar features to that of the M-S3 film, indicating the presence of the hematite phase at the surface. The significant difference between Fe_3_O_4_ and α-Fe_2_O_3_ phases was indicated by the doublet peak of Fe2p_3/2_ and Fe2p_1/2_ of the Fe2p spectral position. The shift in the Fe2p doublet peak by 0.5 eV towards lower binding energy represents the magnetite phase (N-S1) as compared to the hematite phase (N-S2/N-S3); this spectral shift^66^ is presented in ESI Table 2.† The effect of oxygen pressure on N-Fe films was distinguishable using XPS analysis, unlike M-Fe films.

#### 3.1.2. Raman spectroscopic analysis

**Fig. 5:**
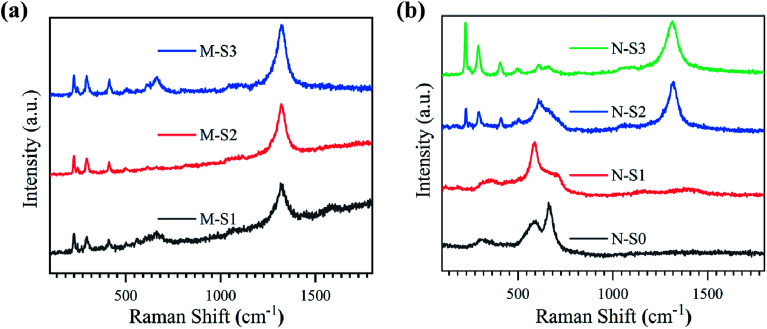
Raman spectra recorded for (a) M-Fe and (b) N-Fe films exposed to PATO (S0-S3).

In order to understand the surface stabilized polymorphs of iron oxide more clearly after the films were processed using PATO, another surface-sensitive spectroscopic technique, *viz.*, Raman spectroscopy, was used. Raman spectroscopic analysis was used to confirm the phases and distinguish them. Fig. 5a and b show the Raman spectra for M-Fe and N-Fe films, respectively, processed by PATO. Table 3 represents the Raman active modes associated with different polymorphs of iron oxide.^69^

**Table 3:** Assignment of Raman active modes representing polymorphs of iron oxide for all M-Fe and N-Fe films processed using PATO (S0-S3)

Sr. no.	Sample name	Raman active modes (cm^−1^)											
		α-Fe_2_O_3_ ^69,73^							γ-Fe_2_O_3_ ^72^			Fe_3_O_4_ ^70,71^	
		A_1g_	E_g_	E_g_	E_g_	A_1g_	T_2g_	E_g_	E_g_	T_2g_	A_1g_	T_2g_	A_1g_
1	M-S1	226	245	292	411	491	501	—	—	—	700	560	663
2	M-S2	226	245	292	411	491	501	612	—	—	—	—	663
3	M-S3	226	245	292	411	491	501	612	—	—	—	560	666
4	N-S0	—	—	—	—	—	—	—	318	—	—	590	661
5	N-S1	—	—	—	—	—	—	—	—	350	—	590	—
6	N-S2	226	244	291	410	498	—	608	Broad peak in the 527–764				
7	N-S3	226	244	291	410	498	—	608	—	—	—	—	661

The Raman spectra in the range of 100–1800 cm^−1^ for the M-Fe films processed by PATO are shown in Fig. 5a. In the case of films M-S1 to M-S3, Raman peaks were observed at 226 cm^−1^ (A_1g_), 245 cm^−1^ (E_g_), 292 cm^−1^ (E_g_), 411 cm^−1^ (E_g_), 491 cm^−1^ (A_1g_) and 501 cm^−1^ (T_2g_) belong to the active modes^67^ of the hematite phase as shown in Table 3. Here, the Raman active mode is indicated in the parentheses. Another peak was observed at 612 cm^−1^ (E_g_) for M-S2 and M-S3, assigned to the hematite phase. The Raman peak observed at 1324 cm^−1^ in these films was due to the two-magnon scattering of hematite, which is not the feature of the magnetite or maghemite phase.^70^ The Raman active modes present in the M-S1 to M-S3 showed hematite as a prominent phase. The careful observation of the spectra depicted a broad peak present in the range of 580–757 cm^−1^ in the case of M-S1, which weakened in the case of the M-S3 film with a broad peak at 666 cm^−1^ in both cases. The weak Raman peak observed at 666 cm^−1^ is associated with the A_1g_ Raman active mode of the magnetite phase. A few more distinct Raman active peaks were observed in the case of M-S1 at 560 cm^−1^ ^71^ and 700 cm^−1^,^72^ which were assigned to the T_2g_ mode of the magnetite and A_1g_ mode of the maghemite phases of iron oxide, respectively. Raman spectroscopic data for M-Fe films are summarized in Table 3, depicting the strong signature of the hematite phase in all the films of M-Fe (M-S1 to M-S3). A weak signature of magnetite and maghemite was seen in the case of M-S1, whereas a weak signature of magnetite only was seen in the M-S2 and M-S3 films.

Fig. 5b represents the Raman spectra for PATO-processed N-Fe films as a function of operating pressure in the ECR plasma reactor due to the increased oxygen flow rate. As depicted in Table 3, Raman peaks are used to identify different polymorphs present at the surface of the N-S0 to N-S3 films. The broad peak at 318 cm^−1^ (E_g_) indicates the Raman-active band of the maghemite phase, and other peaks at 590 cm^−1^ (T_2g_)^74^ and 661 cm^−1^ (A_1g_) belong to the magnetite phase present in the N-S0 film. No peaks were observed at higher wavenumbers, and hence the N-S0 film showed the prominent phase of magnetite with maghemite as a secondary (weak) phase. In the case of N-S1, the first broad peak was shifted to a higher wavenumber as compared to the N-S0 with a peak position at 350 cm^−1^, and the other peak at 701 cm^−1^ (A_1g_) represents the maghemite phase, whereas the peak observed at 590 cm^−1^ (T_2g_) indicated the presence of the magnetite phase. Hence, the N-S1 film consists of magnetite and maghemite phases at the surface, with magnetite as a prominent phase. From the N-S2 film, the Raman active modes observed at 226 cm^−1^, 244 cm^−1^, 291 cm^−1^, 410 cm^−1^, 498 cm^−1^ and 608 cm^−1^ were the signature of the hematite phase. Here, a broad peak was observed at 608 cm^−1^, ranging from 527–764 cm^−1^; the broadening of the peak may be due to the presence of secondary phases like magnetite/maghemite and hence, the N-S2 film does not consist of only the hematite phase at the surface. For the film synthesized at higher oxygen pressure, *i.e.*, the N-S3 film, the Raman active peaks were similar to those of the N-S2 film with an additional weak Raman active band observed at 661 cm^−1^ belonging to the magnetite phase. In the case of the N-S3 film, prominent signatures of a hematite phase were observed. Interestingly, the peak at 1324 cm^−1^ was seen only in N-S2 and N-S3 films, which confirmed the presence of the hematite phase (Fig. 5b). Overall, the Raman analysis reflected strongly about the phases of iron oxide observed after PATO processing, especially in the case of M-Fe. Moreover, Raman analysis suggested the mixed phases observed at the respective surfaces, which was difficult to interpret from XPS analysis.

#### 3.1.3. X-Ray diffraction analysis

**Fig. 6:**
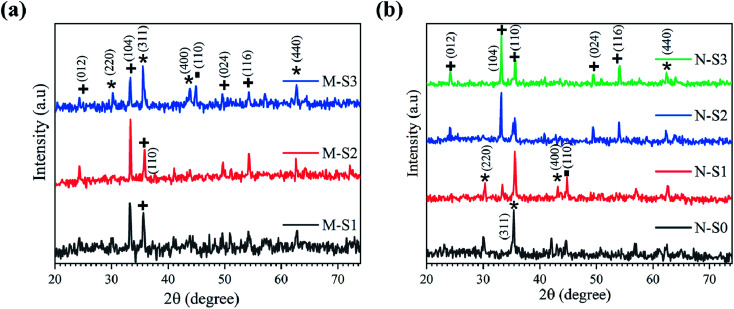
X-ray diffraction patterns recorded for (a) M-S1 to M-S3 of M-Fe and (b) N-S0 to N-S3 of N-Fe films, respectively, exposed to PATO at different oxygen pressure ranges (the symbols represent the JCPDF planes of iron/iron oxide polymorphs; ■: metal Fe (JCPDF card: #851410); *: Fe_3_O_4_/γ-Fe_2_O_3_ (JCPDF card: #861362/#391346); +: α-Fe_2_O_3_ (JCPDF card: 860550)).

Surface analysis is essential to understanding the surface properties; however, the surface and bulk may behave differently in general and more specifically, during the oxidation of iron using the PATO process. Since an oxidative environment is provided to iron powder mixed with ethylcellulose films to grow the polymorphs of iron oxide, it was expected to show different bulk properties. Depending on the temperature and oxygen abundance, different polymorphs of iron oxide, in different parts of the grain were expected to be present. X-ray diffraction was employed to understand the bulk characteristics of the PATO-processed films. Fig. 6 show the X-ray diffraction patterns of M-Fe and N-Fe films recorded after PATO processing. It is clear from the Raman spectroscopic analysis that the M-Fe films consist of hematite as a prominent phase, with a secondary (weak) phase of magnetite/maghemite. The X-ray diffraction lines (Fig. 6a) observed for the films M-S1, M-S2 and M-S3 indicate the formation of the hematite phase. In the case of the M-S2 film, the hematite phase seemed to be the prominent phase, whereas the M-S3 film indicated the prominent phase of magnetite/maghemite and hematite as the secondary phase, which supplements the inferences from Raman analysis (Fig. 5a). Careful analysis of the XRD pattern of the M-S3 film indicated the presence of metallic iron along with the oxide phase. This observation indicates the incomplete oxidation of iron films. Generally, the oxidation of metallic iron takes place in the following sequence, Fe → FeO → Fe_3_O_4_ → γ-Fe_2_O_3_ → α-Fe_2_O_3_. This indicates in the case of the M-S3 film that the oxidation process was incomplete. During the process of the hematite phase formation from iron through magnetite and maghemite, oxygen was liberated. The incomplete reaction resulted in the formation of the mixed-phase at the surface. It was reported that at the temperature of about 300 °C, the magnetite phase was transformed into maghemite, which is the metastable phase.^74,75^ Both magnetite and maghemite possess the spinel cubic crystal structure; therefore, by a topotactic transition, maghemite was formed on top of the magnetite surface. As the temperature increased above 500 °C, the magnetite/maghemite was transformed into the hematite phase and their crystal structures were totally different. The difference in the crystal structure introduced the possibility of stress-induced morphology.

Fig. 6b shows the X-ray diffraction patterns for the N-Fe film analyzed after PATO processing. The N-S0, N-S1, N-S2 and N-S3 films were processed by PATO under oxygen as the plasma-forming gas with flow rates of 5, 15, 30 and 100 sccm, respectively. From Fig. 6b, the X-ray diffraction pattern for the N-S0 film depicts the formation of magnetite (Fe_3_O_4_) as the prominent phase; for the N-S1 film, the diffraction lines show magnetite/maghemite as the prominent phase along with traces of the hematite (α-Fe_2_O_3_) secondary phase. In the case of the N-S2 film, the diffraction lines showed hematite as the prominent phase along with Fe_3_O_4_/γ-Fe_2_O_3_ as the secondary phase. The diffraction lines of the N-S3 film show only hematite as the prominent phase. This trend of phase formations was expected because as the oxygen pressure increased, the iron oxide phase transformation occurred from the magnetite to the hematite phase. From Fig. 6, it is interesting to note that the oxygen pressures of 30 sccm and 100 sccm-processed M-Fe and N-Fe films, *viz.*, M-S1, M-S2 and N-S2, N-S3, showed hematite as a prominent phase. The higher oxygen pressure in the case of M-Fe and lower oxygen pressure in the case of N-Fe showed magnetite as a prominent phase.

#### 3.1.4. Morphological analysis

**Fig. 7:**
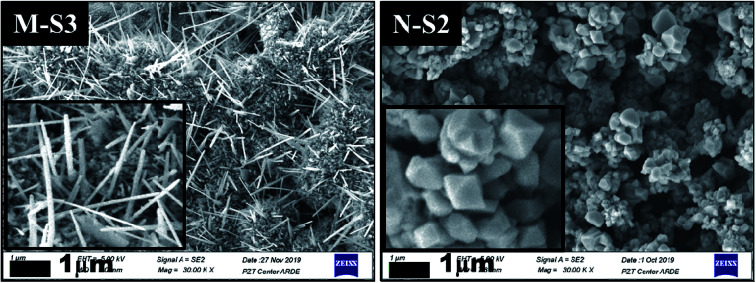
The Field Emission-Scanning Electron Micrographs (FE-SEM) recorded after PATO processing for M-S3 and N-S2 films kept at different processing conditions as mentioned in Table 2, with a 1 μm scale and magnification of ×30 000 (each inset shows the magnified micrograph of the respective film surface; the rest of the films processed under PATO are shown in ESI Fig. 1†).

Fig. 7 shows the FE-SEM micrographs for M-Fe and N-Fe films processed by PATO. Before the PATO process, iron particles were passivated by an ethylcellulose matrix. In the temperature range of 200–500 °C, the ethylcellulose matrix was dissociated and evaporated and at that instant, the oxygen plasma species interacted with iron to form different polymorphs as well as morphologies of iron oxide, depending on the surface reactivity. Fig. 7 indicates the micrographs of M-Fe and N-Fe films processed by PATO at optimized conditions. ESI Fig. 1† shows FE-SEM pictures of all the remaining films of M-Fe and N-Fe processed by PATO in the present study. The distinct morphology of the one-dimensionally grown structure on the surface of iron oxide particles was observed in the case of M-S3. The average length of the one-dimensionally grown structure was found to be in the range of 1.2–1.8 μm with a diameter of ∼0.1 μm. It was also noted that the wire-like growth was not unidirectional. The pyramidal, well-faceted morphology with nano-sized grains was seen in the case of film N-S2. The pyramidal morphology had dimensions in the range of 0.3–0.5 μm.

### 3.2. Phase and morphology tuning

**Fig. 8:**
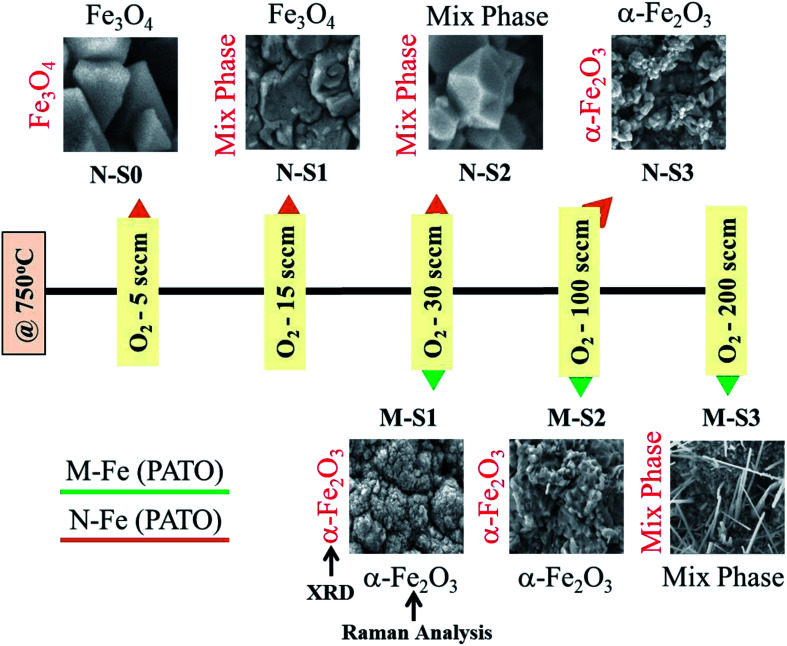
Summary of prominent phases monitored using structural, morphological and Raman analyses of the plasma-assisted thermal oxidation (PATO)-processed M-Fe and N-Fe films kept in different oxygen gas environments.

During the PATO process, the films were exposed to oxygen plasma consisting of plasma species, *viz.*, atomic oxygen and ionic species of oxygen, for 10 min. The key observations based on the experimental results obtained using X-ray diffraction, Raman spectroscopy, X-ray photoelectron spectroscopy and microstructural analysis using FE-SEM are as follows. (i) The structural analysis of M-Fe films indicated the formation of (a) the hematite phase up to the oxygen flow rate of 100 sccm (M-S1) with maghemite/magnetite as a secondary phase, and (b) a mixed phase of hematite (weak phase) and magnetite/maghemite as a prominent phase for an oxygen flow rate of 200 sccm (M-S3). (ii) Raman spectroscopic analysis for M-Fe indicated the presence of hematite as a prominent phase in all M-Fe films with a weak signature of the magnetite as well as the maghemite phase seen in M-S1, and the magnetite phase in the case of M-S2 and M-S3. The observation regarding the M-S3 film coincides with the Shu Nie *et al.*^75^ described model, where hematite was formed during the oxidation of magnetite (4Fe_3_O_4_ + O_2_ → 6Fe_2_O_3_) and there was also a surface step of new magnetite being formed in advance (9Fe_3_O_4_ + 2O_2_ → 12Fe_2_O_3_ + (Fe_3_O_4_)_surface_) during oxygen exposure. (iii) XPS analysis of all three films, M-S1 to M-S3, indicated that they were well defined, and were assigned to the maghemite to hematite phase at the surface. (iv) The morphology of M-S1 was found to be like cauliflower, M-S2 like cabbage, and quite dense one-dimensional growth was observed at the surface of the M-S3 film. On investigating the N-Fe films processed using PATO, (v) the structural analysis based on X-ray diffraction depicted the following. (a) For N-S0, the film formation of a well-defined magnetite phase; (b) for N-S1, the formation of magnetite as a prominent phase along with the signature of hematite; (c) for N-S2, the trend was found to be reversed with hematite as a prominent phase along with the superimposed magnetite/maghemite phase; (d) for N-S3, the complete hematite phase was observed. (vi) Raman spectroscopic analysis indicated the presence of the magnetite phase, in the cases of N-S0 and N-S1, as the prominent phase, with a mixture of maghemite being observed in N-S0 and N-S1. In the case of N-S2, a prominent signature of hematite was observed along with a weak signature of magnetite, and finally, in the case of N-S3, a single hematite phase was observed. (vii) The weakly defined XPS spectrum of N-S0 was due to the magnetite phase, which was found to be strengthened in the case of N-S1. The presence of a satellite peak at 718 eV indicated the hematite phase formation at the surfaces of N-S2 and N-S3. (viii) The morphologies of N-S0 and N-S1 were polygonal, with well-grown grains in the case of N-S1. These grains had pores. The grains of N-S2 were small but well-faceted and grains of N-S3 were small and blunt. Fig. 8 gives the summary of the bulk, surface and morphological properties of M-Fe and N-Fe films processed by PATO under different oxygen pressure conditions.

The structural and morphological properties observed can be explained as follows: usually, the Fe_3_O_4_ (magnetite) phase is formed at a relatively low temperature and in an oxygen-starving atmosphere. Magnetite undergoes oxidation and is transformed into α-Fe_2_O_3_ through γ-Fe_2_O_3_ (maghemite) phase as a result of heat treatment. It is given as 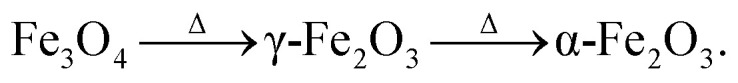
^69^ Fe_3_O_4_ (Fe^2+^ Fe^3+^ Fe^3+^ O_4_^2−^), having a spinel structure, possesses Fe^2+^ and Fe^3+^ ions in a 1 : 2 ratio, out of which the divalent ion is oxidized during the formation process of γ-Fe_2_O_3_ (□ Fe^3+^ Fe^3+^ O_3_^2−^) and a vacancy is created however, the spinel crystal structure is maintained. The formation of α-Fe_2_O_3_ occurs through the oxidation of magnetite/maghemite. Firstly, oxygen species at the surface create iron vacancies and secondly, iron at the core diffuses through the interfaces of magnetite, and these are consumed during the formation of the hematite phase.

In the present case, the formation of different polymorphs of iron oxide is facilitated by the diffusion of oxygen into the iron grain and outward diffusion of iron from the core. During the oxidation process, Fe atoms at the surface gets oxidized and are subjected to a concentration gradient, and hence Fe atoms continuously diffuse out from the core to the surface. Moreover, Fe atoms become ionized during the diffusion process normal to the surface where Fe^2+^/Fe^3+^ diffuse outward (outward diffusion coefficient: 9.7 × 10^−15^ cm^2^ s^−1^) and O^2−^ diffuses inward (inward diffusion coefficient: 5.2 × 10^−16^ cm^2^ s^−1^). This ionic diffusion happens through vacancy exchange rather than direct atom exchange.^76^

In the present case, the role of ethylcellulose, the reactivity of the particles due to different surface to volume ratio, surface temperature and oxygen partial pressure are decisive parameters for the structural and morphological properties. In the case of bare particles (both micron-sized as well as nano-sized), the diffusion of oxygen and iron into each other is quite fast and results in the formation of a stable hematite phase. However, the phase and morphology were found to be different in the presence of ethylcellulose for PATO-processed films. In the case of M-Fe films processed by PATO, the diffusion process facilitated the formation of hematite when the abundance of oxygen was low (*i.e.* up to the flow rate of 100 sccm), whereas for a higher flow rate, *viz.*, 200 sccm, the formation of hematite on magnetite was facilitated.

It is interesting to note the different morphologies observed in the case of M-Fe and N-Fe films. Among the films investigated, whisker growth was observed in the case of M-S3, whereas a faceted morphology was observed in the case of N-S2. The growth of α-Fe_2_O_3_ whiskers followed the same mechanism as that of CuO nanowire formation reported by Yuan *et al.*^77^ The growth of α-Fe_2_O_3_ whiskers is mainly associated with the stress generation and relaxation at the α-Fe_2_O_3_/Fe_3_O_4_ interface. With decreasing oxygen partial pressure towards the Fe core of the grain in the presence of the ethylcellulose matrix, the oxidation rate was slowed down and the Fe_3_O_4_/γ-Fe_2_O_3_ and α-Fe_2_O_3_ interfacial reaction rate was reduced, which resulted in a small stress gradient across the α-Fe_2_O_3_ layer. Influenced by this driving force, the Fe cations diffused along the grain boundary region and were deposited at the bottom of the α-Fe_2_O_3_ phase. The α-Fe_2_O_3_ layer at the surface grows at the expense of the thin Fe_3_O_4_/γ-Fe_2_O_3_ layer at the interface. It was reported that the Pilling and Bedworth ratios (the ratio of the volume of metal oxide to the volume of consumed metal) for FeO, Fe_3_O_4_ and Fe_2_O_3_ were 1.68, 2.10 and 2.14, respectively.^77^ Since the specific volume of Fe_3_O_4_/γ-Fe_2_O_3_ was smaller than that of Fe_2_O_3_, the compressive stresses were generated and accumulated at the bottom of the Fe_2_O_3_ layer. This led to an increased stress gradient and facilitated the outward diffusion and promoted the delivery of Fe cations onto the top of Fe_2_O_3_ grains *via* combined grain boundary and surface diffusion, where the grain surface served as structure templates for the nucleation of Fe_2_O_3_ whiskers.^77^ The drastic growth of the α-Fe_2_O_3_ layer at the surface in the presence of ethylcellulose slowed down the diffusion of oxygen to the core and hence, in the case of M-S3, unreacted Fe traces were observed in the X-ray diffraction pattern. Due to the sufficiently high reactivity of nanoparticles, this feature was not observed in N-Fe films.

### 3.3. Field emission (FE) analysis

**Fig. 9:**
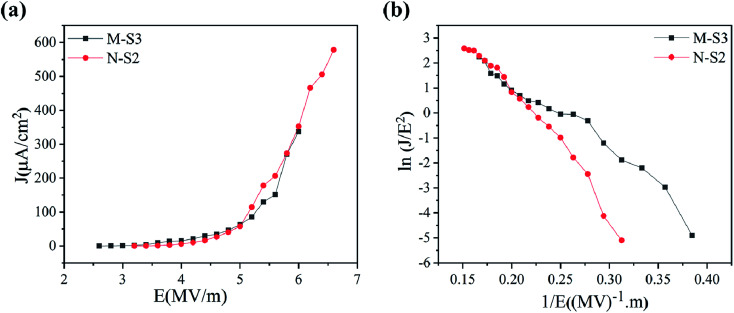
Field emission properties showing (a) the *J*–*V* plot and (b) F-N plot for PATO-processed (M-S3 and N-S2) M-Fe and N-Fe films, respectively.

The field emission electron flux recorded on the phosphorous-coated conducting screen (on the anode) as a function of the applied electric field for M-S3 and N-S2 films is shown in Fig. 9a. It was seen that the turn-on field for the M-S3 film was low as compared to the N-Fe film, whereas the turn-on field for the N-S2 film was low among N-Fe films. Table 4 shows the summary of the field emission properties of the selected films of M-Fe and N-Fe processed by PATO. Among all of these films under investigation, the M-S3 film with moderate whiskers showed a low turn-on field. Nanostructure morphologies, more specifically, one-dimensional nanostructures are expected to show superior field emission properties. However, highly dense emission sites show poor field emission performance.^30^ Li-Chieh *et al.*^30^ reported that the increases in the population density of nanowires per square centimeter indicate the drastic change in the turn-on field, which supports the experimentally observed results in the present case.

**Table 4:** Summary of field electron emission properties such as turn-on field (MV m^−1^), threshold field (MV m^−1^) and maximum current density (μA cm^−2^) for optimized M-Fe and N-Fe films

Sample name	M-S3	N-S0	N-S2	N-S3
Turn-on field 1 μA cm^−2^ (MV m^−1^)	3	5	3.6	3.8
Threshold 10 μA cm^−2^ (MV m^−1^)	3.6	6.8	4.2	4.8
Maximum current density (μA cm^−2^)	337	804	578	260

Field emission properties for N-Fe films are shown in Fig. 9a and b. The PATO-processed N-S2 film having a nanostructured facet (pyramidal morphology) possesses a low turn-on field of 3.6 MV m^−1^ with higher current density (578 μA cm^−2^). The current density in N-S0 was highest in N-Fe because of its surface being stabilized with the magnetite phase, which is a better conductor than the hematite phase. Different morphologies of iron oxide grown over M-Fe and N-Fe-based M-S3 and N-S2 films were found to be good field emitters among the films under investigation.

On comparison of the experimentally observed field emission properties (Table 4) with those reported in the literature as summarized in Table 1, it was seen that the PATO-processed hematite surface had the better field emission performance as compared to that mentioned in Table 1 without any post-processing. Moreover, PATO provides a controlled oxygen environment, green process, processing in a clean environment, less processing time and *in situ* heating to form the desired polymorph of iron oxide at the surface as compared to the methods listed in Table 1. The role of organic matter in encapsulating the iron precursor cannot be avoided. Usually, the iron oxide surface requires prolonged treatment at elevated temperatures to obtain the hematite phase and the typical morphology suitable for field-effect electron emission.^10,25,30,43,44^ From Table 4, PATO-processed films revealed that the method of processing strongly affects the field emission performance; the PATO-processed films with unique morphologies were better than the conventionally processed films. The difference in field emission performance in the PATO-processed M-Fe and N-Fe films was due to the surface-stabilized phase and morphology obtained in a given oxygen environment. Moreover, it is well known that the surface with the higher aspect ratio always demonstrates better field emission behaviour; hence, M-S3 indicated a turn-on field of 3 MV m^−1^ as compared to that of N-S2, showing a wire-like and pyramidal morphology, respectively.

## 4. Conclusion

The present work sheds light on the phase- and morphology-tuning of iron oxide using the Plasma Assisted Thermal Oxidation (PATO) process *via* oxygen ECR plasma, which is the first of its kind. With ECR plasma being cold plasma, a radiation heater was designed, which operates at low pressure and is capable of raising the surface temperature to the desired value within a minute. Low operating pressure, less processing time, rapid heating and controlled oxygen environment are the features of the PATO process used for tuning the specific phase and morphology of iron oxide. For the M-Fe film, the phase transformation from hematite to the mixed iron oxide phase, and the morphology tuning from polygonal to whiskers were observed on increasing the oxygen flow rate from 30–200 sccm. The N-Fe films showed a phase transformation from magnetite to hematite through maghemite with an increased oxygen flow rate from 5–100 sccm. In the PATO-processed iron oxide films, the stress generated at the bottom of α-Fe_2_O_3_ seems to be the driving force for morphological tuning, whereas temperature and partial pressure of oxygen are required for phase tuning. The PATO-processed films M-S3 and N-S2 are comprised of mixed phases of iron oxide as analyzed by XRD and Raman spectra with the α-Fe_2_O_3_ phase at the surface. The field emission study showed that the PATO-processed M-S3 and N-S2 films are the best field emitters. In a nutshell, the present work and its analysis showcase the feasibility of the ECR plasma reactor for tuning the phase and morphology in nanostructure synthesis.

## Conflicts of interest

There are no conflicts to declare.

## Supplementary Material

RA-010-D0RA05410K-s001
