# Supplementary material for: Structural and morphological tuning of iron oxide polymorphs by ECR plasma-assisted thermal oxidation
Source: RSC Adv. 2020 Aug 28;10(53):32088–101. doi: 10.1039/d0ra05410k (PMC9056614; doi:10.1039/d0ra05410k)
Supplement: RA-010-D0RA05410K-s001 [file RA-010-D0RA05410K-s001.pdf]

Electronic Supplementary Material (ESI) for RSC Advances.
This journal is © The Royal Society of Chemistry 2020

Supplementary data

Structural and morphological tuning of iron oxide polymorphs by ECR plasma assisted thermal oxidation

Supriya E. More[a], Suyog A. Raut[a], S. Premkumar[a,b], Somanath R Bhopale[a], Sudha V. Bhoraskar[a], Mahendra A. More[a], Vikas L. Mathe[a]*

[a]*Department of Physics, Savitribai Phule Pune University, Ganeshkhind, Pune 411007, Maharashtra, India.*
[b]*Armament Research and Development Establishment, Pune 411021, Maharashtra, India.*
(Email address: vlmathe@physics.unipune.ac.in / vikasmathe@gmail.com)

1 X-ray Photoelectron spectroscopy:

Supplementary Table 1: XPS analysis of the Fe2p peaks fitted using XPSPEAK software showing fitted peak position (binding energy), FWHM and area under the curve for M-S1, M-S2 and M-S3 films.

		Fe2p									
**M-S1**	Peak (eV)	709.61	710.76	712.23	713.94	718.84	723.26	724.55	725.92	727.42	733.13
	FWHM	1.34	1.69	1.95	2	4.96	1.95	1.95	1.95	1.95	3.45
	Area	3572	6006	4826	3678	1193	2902	2974	2544	1215	1967
		**Fe2p**									
**M-S2**	Peak (eV)	709.49	710.38	711.59	713.1	719.2	723.17	724.57	726.07	728.21	733.75
	FWHM	1.58	1.58	1.68	1.95	2.38	2	2	2	2	4.06
	Area	2253	2860	2098	1783	798	1543	1886	944	392	1490
		**Fe2p**									
**M-S3**	Peak (eV)	709.71	711.14	712.72	714.17	718.63	723.1	724.34	725.64	727.11	732.92
	FWHM	1.71	1.94	1.72	2	3.67	2	2	2	2	4.01
	Area	9084	11625	4697	3053	4073	5379	5363	3047	1286	2865

Supplementary Table 2: XPS analysis of the Fe2p peaks fitted using XPSPEAK software showing fitted peak position (binding energy), FWHM and area under the curve for N-S0, N-S1, N-S2 and N-S3 films.

**N-S0**	**Fe2p**									
	Peak (eV)	709.96	711.55	713.43	719.15	724.29				
	FWHM	2	2	2	2	2.5				
	Area	575	345	139	130	500				
**N-S1**	**Fe2p**									
	Peak (eV)	708.95	710.66	712.19	713.74	722.15	723.83	725.38	727.18	733.48
	FWHM	2	2	2	1.94	2	2	2	2	2
	Area	4112	10193	6359	3350	2261	4503	3563	1324	1000
**N-S2**	**Fe2p**									
	Peak (eV)	709.53	710.79	712.40	713.97	719.3	723.38	724.72	726.35	733.55
	FWHM	2	2	2.15	2	2.4	2	2	2	3.14
	Area	3329	8452	6298	6298	1576	3568	3951	2318	840
**N-S3**	**Fe2p**									
	Peak (eV)	709.54	710.66	712.09	713.76	719.04	723.46	724.77	726.21	733.24
	FWHM	1.53	1.77	1.91	2	3.2	2	2	2	2.93
	Area	3091	7240	5055	3822	3127	3548	3784	2216	694

2 Field Emission Scanning Electron Microscopy (FE-SEM):

Supplementary Fig. 1 includes FE-SEM micrographs of the films M-S1, M-S2, N-S0, N-S1, and N-S3 processed by PATO. The cauliflower like morphology is seen for the M-S1 film and agglomerated sheet like morphology for M-S2 film. Here, N-S0 film shows irregular morphology with wide size distribution. Film N-S1 show grain growth with respect to N-S0 and the grains possesses small pores in it. N-S3 consists of mixed morphology in terms of size distribution as well as shapes.

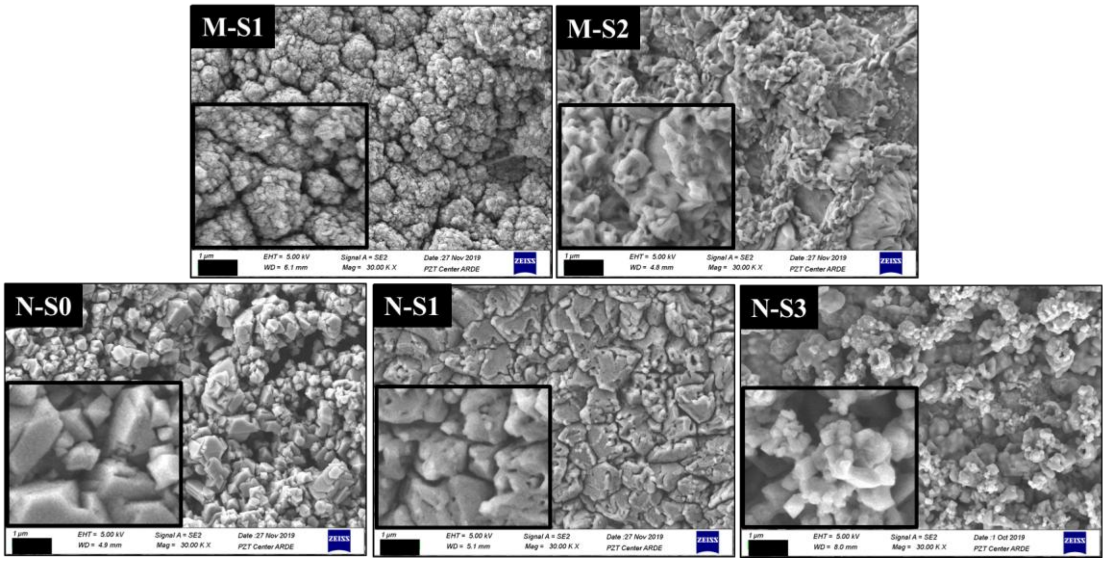

Supplementary Figure 1. The Field emission - Scanning Electron Micrographs (FE-SEM) recorded after PATO processed for M-S1/S2 and N-S0/1/3 films kept at different processing condition with a 1[illegible]m scale and magnification of x30000. (Every inset shows magnified micrograph of respective sample surface)
